# A *BAP1* Mutation in a Danish Family Predisposes to Uveal Melanoma and Other Cancers

**DOI:** 10.1371/journal.pone.0072144

**Published:** 2013-08-19

**Authors:** Lauren G. Aoude, Karin Wadt, Anders Bojesen, Dorthe Crüger, Åke Borg, Jeffrey M. Trent, Kevin M. Brown, Anne-Marie Gerdes, Göran Jönsson, Nicholas K. Hayward

**Affiliations:** 1 Oncogenomics Laboratory, Queensland Institute of Medical Research, Brisbane, Queensland, Australia; 2 University of Queensland, Brisbane, Queensland, Australia; 3 Department of Clinical Genetics, Rigshospitalet, Copenhagen, Denmark; 4 Department of Clinical Genetics, Vejle Hospital, Vejle, Denmark; 5 Department of Clinical Sciences Lund, Division of Oncology, Lund University, Lund, Sweden; 6 Translational Genomics Research Institute, Phoenix, Arizona, United States of America; 7 Laboratory of Translational Genomics, National Cancer Institute, Bethesda, Maryland, United States of America; University of Connecticut Health Center, United States of America

## Abstract

Truncating germline mutations in the tumor suppressor gene BRCA-1 associated protein-1 (*BAP1*) have been reported in families predisposed to developing a wide range of different cancer types including uveal melanoma and cutaneous melanoma. There has also been an association between amelanotic tumor development and germline *BAP1* mutation suggesting a possible phenotypic characteristic of *BAP1* mutation carriers. Though there have been many types of cancer associated with germline *BAP1* mutation, the full spectrum of disease association is yet to be ascertained. Here we describe a Danish family with predominantly uveal melanoma but also a range of other tumor types including lung, neuroendocrine, stomach, and breast cancer; as well as pigmented skin lesions. Whole-exome sequencing identified a *BAP1* splice mutation located at c.581-2A>G, which leads to a premature truncation of *BAP1* in an individual with uveal melanoma. This mutation was carried by several other family members with melanoma or various cancers. The finding expands on the growing profile of *BAP1* as an important uveal and cutaneous melanoma tumor suppressor gene and implicates its involvement in the development of lung, and stomach cancer.

## Introduction

BRCA-1 associated protein-1 (*BAP1*) is a tumor suppressor gene located on chromosome 3, a region that has been linked to uveal melanoma (UMM). In the first report to make an association between *BAP1* and UMM Harbour and colleagues used a next-generation sequencing approach to exome-sequence two tumors from patients with metastatic UMM [1]. *BAP1* was found to be the only gene that was mutated on chromosome 3 in each of these samples and both mutations led to truncation of the protein. They next interrogated 57 UMM tumors using Sanger sequencing and found that *BAP1* mutations occurred predominantly in tumors that had metastasized. Overall, 26/31 of the metastasizing (high risk) tumors had inactivating *BAP1* somatic mutations compared to only 1/26 of the low risk (non-metastatic) group. For the 20 samples with a matched normal DNA sample, almost all *BAP1* mutations were found to be acquired somatically, the exception being a single case with a matching germline frameshift mutation. This mutation introduced the possibility that *BAP1* defects could predispose to UMM. Leading on from the study by Harbour et. al., several groups have looked at the risk of disease conferred by germline *BAP1* mutation. Testa and colleagues investigated *BAP1* mutations in two American families presenting with mesothelioma and who had no contact with any of the known environmental risk factors for this disease [2]. They found two different frameshift mutations in *BAP1* responsible for the elevated risk of mesothelioma in these individuals. Interestingly, these families also presented with a range of cancers that included UMM and cutaneous melanoma (CMM). A second study also supported these findings by interrogating germline DNA from a family that presented with mesothelioma, meningioma and UMM [3]. In this family a nonsense mutation caused premature truncation of the BAP1 protein in carriers. A report by Wiesner showed *BAP1* mutations in two families that had an autosomal dominant syndrome characterized by UMM, CMM and atypical benign melanocytic nevi [4]. The two families presented with unique frameshift mutations that co-segregated with the disease phenotype. In a follow up report, Wiesner and colleagues identified a third family with a *BAP1* mutation that co-segregated with mesothelioma and also showed evidence of a melanocytic lesion in a mutation carrier [5]. Other groups have also described *BAP1* mutations in individuals presenting with atypical intradermal tumors, proposing that these lesions may be a phenotypic characteristic of *BAP1* mutation carriers [6], [7]. A recent study of Portuguese siblings with a rare subtype of epithelioid mesothelioma uncovered a germline *BAP1* mutation as the possible cause of the only known familial clustering of well-differentiated papillary mesothelioma. Notably one of the siblings also developed UMM [8]. Recently a cryptic splice mutation in *BAP1* was found to co-segregate in a Danish family with UMM, paraganglioma and atypical CMM [9]. *BAP1* mutation has also been implicated in the development of renal cell carcinoma and some of the relatives of these cases have had UMM or CMM also [10]. As evidenced by the literature, there is a large tumor spectrum that appears to accompany *BAP1* germline mutation.

**Figure 1:**
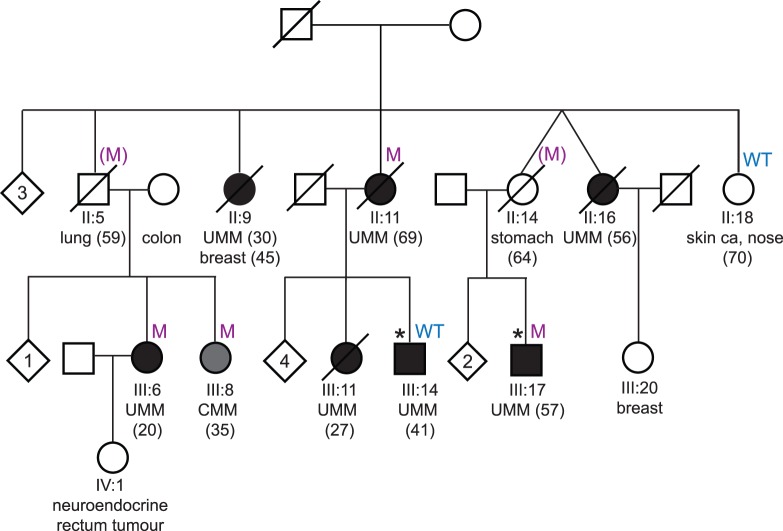
Co-segregation analysis of a *BAP1* splice mutation in a Danish family. In the pedigree individuals that have uveal melanoma (UMM) are represented by black circles (female) or boxes (male) and individuals with cutaneous melanoma (CMM) are indicated by grey circles or boxes. The age of diagnosis of each melanoma is indicated in brackets. A line through a symbol indicates that the person is now deceased. If a person carries the *BAP1* splice mutation it is indicated by an ‘M’ and if they are wild type for this variant it is indicated with a ‘WT’. Where the mutation status is indicated in brackets, the person is a presumed obligate carrier, ‘(M)’. Other cancer types are also indicated in the pedigree with the age of diagnosis in brackets. Asterisks indicate the two individuals that were exome sequenced. Unaffected siblings are represented by diamonds, with the number in the centre indicating the number of people.

In this paper we report a Danish family predisposed to developing UMM predominantly but also presents with a wide range of seemingly unrelated tumors. There are 7 family members that have had UMM, with the youngest age of onset being 20. One developed UMM at the age of 30 and later went on to develop breast cancer at age 45. There is an individual with lung cancer who had two children that developed melanoma, one UMM and the other CMM. Another individual developed UMM at the age of 69 and has two children that were diagnosed with UMM at ages 27 and 41. There is a single instance of diffuse infiltrating gastric cancer and this individual also had a child with UMM, diagnosed at age 57. Finally, another case of UMM was diagnosed at age 56. Refer to Figure 1 for a family pedigree which identifies the cancer history of each individual.

Aside from the striking incidence of cancer, this family presents with other clinical features that have become indications of *BAP1* mutation. Four of the five individuals with UMM died of liver metastasis derived from the UMM. Individual III:6, who was diagnosed with UMM at age 20, had an isolated iris melanoma and later developed three basal cell carcinomas. She is currently 61 years old and well. Individual III:8, with CMM diagnosed at 35 had a tumor localized in the occipital region, which most likely was a primary CMM but metastasis could not be excluded. She died at 39 years of age with disseminated CMM.

## Methods

### Ethics Statement

Ethics approval for this project was granted by the Human Research Ethics Committees of the Queensland Institute of Medical Research and Lund University. Written consent was obtained for each individual.

### Samples

Whole blood was collected from disease affected individuals in dense uveal melanoma families. Genomic DNA was extracted using standard methods. DNA samples were then used for exome and Sanger sequencing experiments.

### Whole-Exome Sequencing

In order to find disease-associated variants, whole-exome sequencing was carried out on key individuals representative of the tumor burden in this family. Two UMM cases that were three meioses apart (III:14 and III:17, Figure 1) were selected and sequencing was carried out using an Illumina Hiseq 2000 and an Illumina TruSeq Exome Enrichment Kit. The reads that were generated were mapped to reference genome UCSC hg19 using the BWA alignment algorithm [11]. SAMTOOLS was then used to detect the SNPs and indels [12]. This process generates large volumes of data and in order to sculpt the long list of resulting variants into a manageable panel of variants several filtering criteria were applied to the data set. Firstly, any variants that were seen in dbSNP (build 132) or reported by the 1000 Genomes Project (February 2012) were filtered out, leaving only novel variants for consideration. Secondly, synonymous changes were removed, leaving only the protein-altering variants. A cut off of 40 was then applied to the quality score returned for each variant in order to limit the false positive rate amongst the called variants. A 20% cut off was also applied to the number of alternate reads, that is, if a variant did not have a high enough percentage of reads for the alternate allele then it was removed from consideration. What remained after this filtering process were 140 novel variants for individual III:14 and 145 novel variants for individual III:17. Exome data from these individuals may be made available for research purposes upon request to the authors.

**Figure 2:**
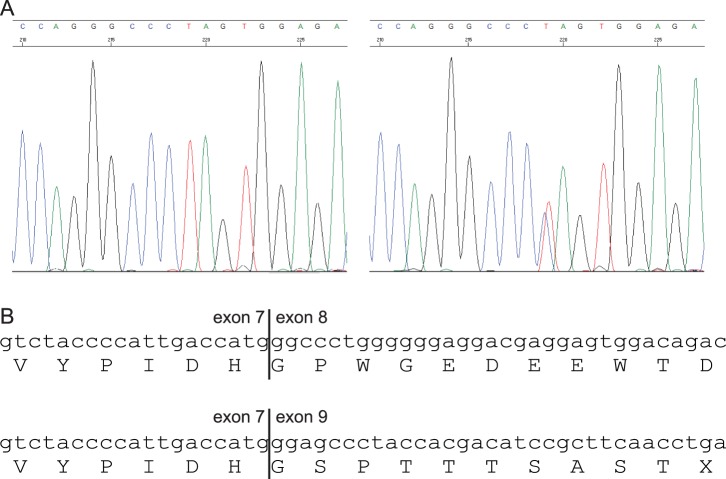
Sanger sequencing trace and amino acid alignment showing the truncated BAP1 protein. (A) The left panel shows the wild type chromatogram while the right panel shows that of the *BAP1* splice mutation. (B) Wild type nucleotide and amino acid sequences are shown in the upper panel while the lower panel shows part of the truncated BAP1 protein resulting from the loss of exon 8.

Exome-sequencing uncovered a *BAP1* splice mutation, which results in the premature truncation of the protein, in one (III:17) of the two individuals and was seen as a likely candidate responsible for UMM susceptibility due to the supporting literature. The result was verified in the individual using Sanger sequencing (refer to Figure 2). Following this, co-segregation analysis was carried out to determine how well the variant segregated with disease in the family and whether it was present in individuals with different cancer types. Every available blood DNA sample from this family was screened for the variant. For individual II:11 DNA was obtained from tumor tissue. Additionally, a new blood sample was obtained from individual III:14 since the initial sample did not show presence of a germline *BAP1* mutation. The second blood sample was screened for the specific mutation in *BAP1* using Sanger sequencing.

### RT-PCR

**Figure 3:**
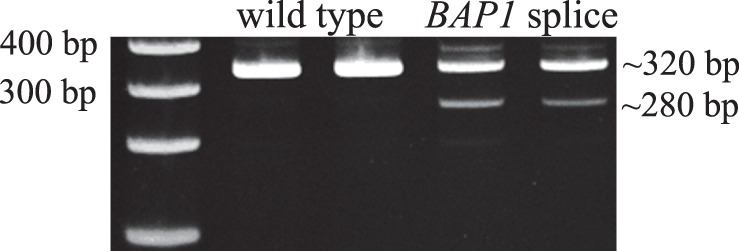
RT-PCR showing the aberrantly spliced *BAP1* transcript. From the left, the first lane shows a size marker, the next two lanes show wild type *BAP1* RT-PCR products, and the last two lanes show the aberrantly spliced product resulting from the c.581-2A>G mutation.

RT-PCR was performed on cDNA from one carrier (III:6) to verify that the *BAP1* mutation was indeed a splicing variant. RNA was converted to cDNA using Superscript II (Life technologies) and primers flanking the exons affected by the putative splice site were designed. Subsequently, PCR products were run on an acrylamide gel (refer to Figure 3).

### Case-Control Genotyping

To more fully understand the role that this variant could play in melanoma risk an Australian case-control sample was genotyped for this novel variant. A Sequenom iPLEX was run on 1655 affected probands from families that had either CMM, UMM or a combination of both, and 1596 controls. The sample set included a wide cross-section of cases with varied histories of melanoma. Some of the probands are from dense melanoma families while others are sporadic cases. This cohort also includes cases with a wide spectrum of ages of onset, which range from childhood disease to late onset melanoma.

## Results

Whole-exome-sequencing identified a *BAP1* splice mutation (c.581-2A>G) in an individual with UMM from a Danish family predisposed to developing UMM as well as a host of other cancers. Co-segregation analysis showed that three out of the four UMM cases with DNA available for research purposes were carriers of this mutation, which causes premature truncation of *BAP1*. Additionally, there were four other individuals with different cancer types that also carried the mutation. These cancers were CMM, lung cancer and stomach cancer. To confirm that individual III:14 was wildtype for the *BAP1* mutation a newly obtained blood sample was screened. Indeed, the analysis confirmed the absence of *BAP1* mutation suggesting that this case is a phenocopy. Loss of heterozygosity (LOH) of the *BAP1* mutation was also observed in DNA from tumor tissue from II:11.

Consistent with data from the 1000 Genomes Project, genotyping results showed that this variant was only present in the current family, that is only 1/1655 melanoma probands carried the variant and 0/1596 controls. Data from the NHLBI GO Exome Sequencing Project (ESP) also reflects this finding as this splicing variant is not found in any of the 6500 individuals sequenced from the USA [13]. Exome data from 200 healthy Danish controls was also examined for *BAP1* mutation and no protein altering variant was seen [14]. This suggests that the individuals described here most likely carry a mutation ‘private’ to this family.

## Discussion

There have been several cancer types associated with *BAP1* germline mutations but the full spectrum of tumor susceptibility is still to be ascertained. We report here a Danish family with UMM and pigmented skin lesions, as well as lung, neuroendocrine, stomach, and breast cancers. Whole-exome sequencing of a UMM case identified a *BAP1* splice mutation (c.581-2A>G), which leads to premature truncation of *BAP1*. This frameshift aberration is consistent with published germline mutations seen in other families predisposed to UMM. In keeping with other Scandinavian germline *BAP1* carriers, we also observe a younger age at diagnosis of UMM (Table 1). In the present and the previously published Scandinavian families, the mean age at diagnosis of UMM was 42 years for the nine affected individuals, compared to 53 years for UMM carriers in non-Scandinavian families (Table 1 and references therein). This compares to a median age at diagnosis for UMM of between 58-62 years in population-based sporadic cases in Scandinavia [15] and the United States [16]. The role of *BAP1* as a tumor suppressor gene was further supported by LOH of the wild type allele in UMM tumor tissue of II:11. The wild type individual that is affected with UMM shows that in dense families it is possible to observe phenocopies even with a tumor type as rare as UMM. The exome sequence data of the person with UMM and wild type *BAP1* did not reveal mutations in other known cancer predisposition genes.

**Table 1:** Clinical phenotypes of current and published UMM cases or their families with *BAP1* mutations.

	Scandinavian families			non-Scandinavian families								
	CurrentFamily	Wadtet al [9]	Höiomet al [17]	Testaet al [2]	Wiesneret al [4], [5]	Njauwet al [18]	Abdel-Rahmanet al [3]	Popovaet al [10]	Ribeiroet al [8]	Scandinavian	non-Scandinavian	total
number UMM cases in family	7	3	3	4	2	8	3	14	1	13	32	45
cases with genotype data	4	3	3	3	2	8	2	8	1	10	24	34
cases with *BAP1* mutation	3	3	3	3	2	8	2	8	1	9	24	33
cases with metastatic disease	3^a^	2^b^	3	0^c^	na	4^d^	na	na	0	8	4	12
ages of onset of carriers	20, 57, 69	18, 46, 62	16, 39, 44	55, 59, 63	44, 72	37, 51, 53, 55, 57, 58, 62,*	na	35, 44, 44, 48,52, 53, 53, 57	56	∧	∼	§
mean age of onset of carriers	49	42	33	59	58	54	na	49	56	42	53	50

na is not available; a has been observed for 41 years without metastatic disease; b has been observed for 16 years without metastatic disease; c has been observed for 1, 4 and 8 years without metastatic disease; d has no information regarding the 4 other UMM cases;

* refers to UMM case with unknown age of onset;

∧ refers to values from columns 1–3;

∼ refers to values from columns 4–9;

§ refers to values from columns 1–9.

In summary, this finding expands on the growing profile of *BAP1* as an important uveal and cutaneous melanoma suppressor gene. The family described here also had a variety of other cancer types, some of which have been implicated with *BAP1* mutation (lung) and some that have not (stomach, neuroendocrine). To assess the possible involvement of *BAP1* in predisposition to these non-melanoma cancers and to more fully understand the spectrum of disease associated with such mutations, large population-based studies are required.

## Supporting Information

Methods S1 **Addition information on whole-exome sequencing, Sanger sequencing and Sequenom iPLEX methods.**
(DOCX)
