# Supplementary material for: A BAP1 Mutation in a Danish Family Predisposes to Uveal Melanoma and Other Cancers
Source: PLoS One. 2013 Aug 19;8(8):e72144. doi: 10.1371/journal.pone.0072144 (PMC3747051; doi:10.1371/journal.pone.0072144)
Supplement: Methods S1 — Addition information on whole-exome sequencing, Sanger sequencing and Sequenom iPLEX methods. (DOCX) [file pone.0072144.s001.docx]

**Supplementary Information**

Methods

Whole-exome Sequencing

Whole-exome sequencing was carried out on two members of a Danish family predisposed to uveal melanoma. Both individuals were affected with UMM and had a family history of disease that is not limited to UMM but also includes several other cancer types. The sequencing was performed on the Illumina HiSeq 2000 using the Illumina TruSeq Exome Enrichment Kit. The sequencing was outsourced to Axeq Technologies in Seoul, South Korea. The sequencing was paired-end and had a median coverage of 100x. Sequence data were mapped to the Human Genome build 19 (hg19). Any variant that was found in dbSNP or the 1000 Genomes project was filtered out leaving only the novel mutations. By applying cut offs to the quality score and the rate of mutant calls for each variant, we were left with novel 140 variants in III:14 and 145 novel variants in III:17 for follow up.

Sanger Sequencing

Sanger sequencing was used to validate the *BAP1* splice mutation (c.581-2A>G) identified through whole-exome sequencing. A PCR reaction was run using 0.5 units of Qiagen HotStarTaq, 2ul of 10x PCR Buffer, 4ul of 5x Q-Solution, 1.6ul of 2.5mM dNTP mix, 1mM each of the forward and reverse primers (CCTGGCTCAACTGCTCTTCT and GCCCAGGCAGGAAATAAGAC respectively) and 15ng of genomic DNA, made up to a final reaction volume of 20ul.

The PCR was run on a Biorad Thermocycler according to Qiagen PCR protocol. It is initiated with a 5 minute activation step at a temperature of 95^o^C, followed by a 3-step cycling process which is repeated 35 times. The cycle starts with a denaturation step at a temperature of 94^o^C for 45 seconds followed by an annealing step at 65^o^C for 45 seconds and then an extension step at 72^o^C for 45 seconds. This 3-step process is repeated with a 65^o^C annealing temperature for two cycles and then steps down in 2^o^C increments until it reaches 57^o^C where the temperature remains until the full 35 cycles are complete. A final 10 minute extension at 72^o^C finalises the PCR run.

The crude PCR product was sent to Functional Biosciences, Wisconsin, USA, to be cleaned up and run on a sequencer. The AB1 files were analysed using an online chromatogram comparison tool, Multiple SeqDoC (http://research.imb.uq.edu.au/seqdoc/multi.html) which is curated by the University of Queensland. The traces were also analysed using chromatogram viewer, Chromas (version 1.45).

Sequenom iPLEX

The *BAP1* c.581-2A>G splice variant was multiplexed using the Sequenom iPLEX gold system which allows up to 36 variants to be genotyped concurrently using 10 ng of genomic DNA. The iPLEX Gold PCR amplification reactions were carried out in 384-well plates according to supplier protocol and then transferred onto SpectroCHIP arrays using a MassARRAY Nanodispenser. The chips were then transferred to a Sequenom mass spectrometer where the data were generated. The results were analysed using Typer Analyzer software 4.0.
